# Pharmacokinetic and Pharmacogenetic Associations with Dolutegravir Neuropsychiatric Adverse Events in an African Population

**DOI:** 10.1093/jac/dkac290

**Published:** 2022-08-29

**Authors:** Rulan Griesel, Phumla Sinxadi, Aida Kawuma, John Joska, Simiso Sokhela, Godspower Akpomiemie, Francois Venter, Paolo Denti, David W. Haas, Gary Maartens

**Affiliations:** 1 Division of Clinical Pharmacology, Department of Medicine, University of Cape Town, Cape Town, South Africa; 2 Wellcome Centre for Infectious Diseases Research in Africa, Institute of Infectious Disease and Molecular Medicine, University of Cape Town, Cape Town, South Africa; 3 HIV Mental Health Research Unit, Division of Neuropsychiatry, Department of Psychiatry and Mental Health, University of Cape Town; 4 Ezintsha, Wits Reproductive Health and HIV Institute, Faculty of Health Sciences, University of the Witwatersrand, Johannesburg, South Africa; 5 Department of Medicine, Vanderbilt University Medical Center, Nashville, Tennessee, USA; 6 Department of Internal Medicine, Meharry Medical College, Nashville, Tennessee, USA

## Abstract

**Background:**

Dolutegravir has been associated with neuropsychiatric adverse events (NPAEs), but relationships between dolutegravir concentrations and NPAEs are unclear.

**Objectives:**

To determine in an African population whether a concentration-response relationship exists between dolutegravir and treatment-emergent NPAEs, and whether selected loss-of-function polymorphisms in genes encoding UDP-glucuronosyltransferase-1A1 (the major metabolising enzyme for dolutegravir) and organic cation transporter-2 (involved in neurotransmitter transport and inhibited by dolutegravir) are associations with NPAEs.

**Methods:**

Antiretroviral therapy-naïve participants randomised to dolutegravir-based therapy in the ADVANCE study were enrolled into a pharmacokinetic sub-study. Primary outcome was change in mental health screening (modified mini screen [MMS]) and sleep quality from baseline to weeks 4, 12, and 24. Dolutegravir exposure was estimated using a population pharmacokinetic model. Polymorphisms analyzed were *UGT1A1* rs887829 and *SLC22A2* rs316019.

**Results:**

Data from 464 participants were available for pharmacokinetic analyses and 301 for genetic analyses. By multivariable linear regression, higher dolutegravir exposure was associated with worsening sleep quality only at week 12 (coefficient = -0.854 [95% CI -1.703 to -0.005], P = 0.049, but with improved MMS score at weeks 12 and 24 (coefficient = -1.255 [95% CI -2.250 to -0.261], P = 0.013 and coefficient = -1.199 [95% CI -2.030 to -0.368], P = 0.005, respectively). The *UGT1A1* and *SLC22A2* polymorphisms were not associated with change in MMS score or sleep quality.

**Conclusions:**

Only at week 12 did we find evidence of a relationship between dolutegravir exposure and worsening sleep quality. However, higher dolutegravir exposure was associated with improved MMS scores, suggesting a possible beneficial effect.

## Introduction

The WHO recommends dolutegravir-based ART as preferred first- and second-line treatment for persons living with HIV (PLWH).^1^ Dolutegravir has been associated with neuropsychiatric adverse events (NPAEs), including insomnia, dizziness, anxiety, depression, headaches, and cognitive impairment. NPAEs associated with dolutegravir are generally mild, as illustrated by a <1% rate of discontinuation for NPAEs in the first year in randomised controlled trials.^2–6^ However, post-marketing cohort studies have reported higher incidences of discontinuation due to dolutegravir-related NPAEs, ranging from 1.4 to 7.2%.^7^

In a German cohort of 985 participants, NPAEs leading to dolutegravir discontinuation within 12 months were observed more frequently among the elderly and women.^8^ A population pharmacokinetic model of dolutegravir among ART-naïve PLWH reported a higher oral bioavailability among women than men, suggesting that there may be a concentration-response relationship for dolutegravir-related NPAEs.^9^ However, evidence supporting this is limited and contradictory. A Japanese study of 107 participants reported that median dolutegravir trough plasma concentrations were higher among participants with NPAEs than those without.^10^ A pharmacokinetic study of 40 PLWH switched to dolutegravir-based ART found no association between dolutegravir pharmacokinetic parameters and sleep or cognition changes over 180 days of follow-up,^11^ and a retrospective case-control study found no association between dolutegravir plasma concentrations and risk of discontinuation due to NPAEs.^12^ Finally, a dolutegravir population pharmacokinetic model derived from three phase 2 and 3 studies failed to show any relationship between dolutegravir exposure and selected adverse event safety endpoints (nausea, diarrhoea, and headache).^9^

Limited evidence from pharmacogenetic studies suggests that there might be a concentration-response relationship between dolutegravir and NPAEs. Dolutegravir is predominantly metabolised by uridine diphosphate (UDP)-glucuronosyltransferase 1A1 (UGT1A1).^13^ Loss of function polymorphisms (UGT1A*28 [rs 3064744 (TA)_7_] and UGT1A1*6 [rs 4148323 G→A]) were associated with a higher incidence of dolutegravir-related NPAEs.^10^ An Italian study reported that a loss of function polymorphism in the *SLC22A2* gene (rs316019 C→A) was associated with NPAEs on dolutegravir-based therapy.^14^ The *SLC22A2* gene encodes for organic cation transporter-2 (OCT2), which is involved in CNS monoamine clearance and is inhibited by dolutegravir at clinically observed concentrations.^13^

Current evidence is contradictory on whether a concentration-response relationship exists for dolutegravir-related NPAEs. Most research on dolutegravir-related NPAEs have not involved people of African ancestry, who have a higher allele frequency of the *UGT1A1* rs3064744 loss-of-function polymorphism than people of European ancestry.^15^ We aimed to establish whether dolutegravir-related NPAEs are associated with dolutegravir plasma concentrations. We also assessed whether polymorphisms associated with increased plasma dolutegravir concentrations and decreased CNS monoamine clearance are associated with dolutegravir-related NPAEs in individuals of African ancestry. We used data obtained from ART-naïve participants who were randomised to initiate dolutegravir-based treatment in the ADVANCE study.^16^

## Methods

### Study Design and Participants

The ADVANCE study was an open label randomised controlled trial conducted in Johannesburg, South Africa.^16^ ART-naïve participants were randomised to one of three arms: 1) dolutegravir, emtricitabine, and tenofovir disoproxil fumarate; 2) dolutegravir, emtricitabine, and tenofovir alafenamide; and 3) efavirenz, emtricitabine, and tenofovir disoproxil fumarate. Trial inclusion criteria included age ≥12 years, no ART use in the previous 6 months, creatinine clearance of >60 mL/minute, and HIV-1 RNA ≥500 copies/mL.

This pharmacology sub-study had the following inclusion criteria: adults (age ≥18 years); participants with sparse dolutegravir plasma samples available or consented to genetic testing; baseline, week 4, 12, and 24 sleep quality and mental health assessments. Exclusion criteria for this sub-study were participants with dolutegravir concentrations below the lower limit of quantification (LLOQ) of the assay, or values greater than four standard deviations of the participant mean (indicating improbable dose sampling times), women who became pregnant during the first 24 weeks of follow-up, and participants who received rifampicin-based antituberculosis therapy during the first 24 weeks of follow-up.

### Psychiatric and Sleep Quality Asssessment

Participants in the ADVANCE trial had a modified mini screen (MMS) mental health assessment at baseline, and at weeks 4, 12, and 24 of follow-up. The MMS is a 22-item scoring questionnaire that covers current symptoms for major depression, dysthymia, suicidality, hypomania, panic, agoraphobia, social phobia, obsessive compulsive disorder, post-traumatic stress disorder (PTSD), psychosis, and generalised anxiety (Supplemental Figure 1). The score has been validated as a screening tool for mental health concerns in addiction, corrections and social service settings.^17,18^ The MMS has been utilised to identify mental health concerns that require more in-depth assessment. A score of 6 or more identifies patients with a moderate likelihood of mental illness, and to be considered for a detailed diagnostic interview. A score of 9 indicates a high likelihood of mental illness and warrants immediate referral for assessment.^19,20^ We removed two questions related to PTSD, as these involved traumatic events, which could be present prior to enrolment. At specified visits, participants were asked to rate the average quality of their sleep in a preceding 4 weeks using a Likert scale from 0 – 10 (0 worst possible quality of sleep, 10 best possible quality of sleep). Sleep quality was assessed at baseline, and at weeks 4, 12, and 24 of follow-up.

### Drug Concentration Analyses

Dolutegravir plasma concentrations were determined by a validated liquid chromatography tandem mass spectrometry assay.^21^ All assays were performed at the Division of Clinical Pharmacology, University of Cape Town, Cape Town, South Africa. The laboratory participated in the Clinical Pharmacology Quality Assurance (CPQA) external quality control program under a contract with the Division of AIDS of the National Institute of Allergy and Infectious Diseases.

### Pharmacokinetic Determinants and Modelling

An intensively sampled pharmacokinetic sub-study (n = 41) nested within the ADVANCE trial was used to develop a population pharmacokinetic model of dolutegravir.^21^ Four-hundred-and-thirty-one participants had sparse sampling of dolutegravir at weeks 24 and 48, with self-reported time of prior dose. The population pharmacokinetic model was used to produce individual estimates of steady-state area under the concentration-time curve over 24 hours (AUC_0-24_) for 472 participants (including intensively sampled data).

### Determination and Characterisation of Genetic Polymorphisms

Whole blood labelled with coded identifiers was stored and DNA extraction performed using the salting out method as described elsewhere.^22^ Genotyping with the Illumina Infinium Multi-Ethnic Global BeadChip (MEGA^EX^) was done at Vanderbilt Technologies for Advanced Genomics (VANTAGE) in Nashville, Tennessee, USA. Post-genotype quality control included sex checks, call rates by marker and sample, identity by descent (IDB) plots, assessment for batch effects, concordance between duplicate samples, and HapMap controls.

Quality control steps were performed using PLINK version 1.9.^23–25^ Genotyping efficiency per participant was >95% in all samples. Markers with genotyping efficiency <95% were excluded, as were those with minor allele frequencies (MAF) <5%. We excluded 21 samples with overall genotyping call rates <95%. After quality control, data were imputed using the TOPMed reference panel after transforming to genome build 38 using liftOver and stratification by chromosome to parallelise the imputation process.^24,25^ For each chromosome in each phase, 100% concordance with genotyped data was assessed. Polymorphisms with imputation scores <0.3, genotyping call rates <99%, MAF <0.05, or Hardy-Weinberg Equilibrium (HWE) p-values <1.0x10^-8^ were excluded. To control for population stratification, we used Eigenstrat/Eigensoft package 6.0.1 to estimate principal components.

The *UGT1A1* rs4148323 locus was monomorphic in our cohort, thus excluded. The UGT1A1*28 promoter TA_n_ dinucleotide repeat (which confers Gilbert trait) was not directly genotyped, as it known to be in strong linkage disequilibrium with the *UGT1A1* rs887829 T allele.^26^ For these analyses, we extracted from genome-wide genotype data two targeted polymorphisms relevant to dolutegravir metabolism (*UGT1A1* rs887829 C→T) and transport of neurotransmitters (*SLC22A2* rs316019 C→A). Participants with genotype data were classified as normal (homozygous CC for *UGT1A1* rs887829 and *SLC22A2* rs316019), intermediate (heterozygous CT and CA, for *UGT1A1* rs887829 and *SLC22A2* rs316019, respectively) and poor (homozygous TT and AA, for *UGT1A1* rs887829 and *SLC22A2* rs316019, respectively).

### Statistical Analysis

Statistical analyses were performed using Stata (version 16.0; StataCorp: Stata Statistical Software, College Station, Texas, USA). Graphs were made using GraphPad Prism (version 9.0; GraphPad Software, San Diego, California, USA). Medians with IQR were used to describe continuous variables and proportions to describe categorical data.

Outcome variables included change in MMS score from baseline to weeks 4, 12, and 24, and change in sleep quality from baseline to weeks 4, 12, and 24. The primary study objective was assessment of associations between the two outcome variables and dolutegravir AUC_0-24_ estimates. Secondary objectives included assessment of associations between the outcome variables, *UGT1A1* rs887829 and *SLC22A2* rs316019.

We used Spearman’s rank-order correlation (*r*_s_) to assess dolutegravir AUC_0-24_ estimates with change in MMS score and change in sleep quality from baseline to weeks 4, 12, and 24. Box plots, grouped by *UGT1A1* or *SLC22A2* genotype, were used to display change in both outcome variables (change in MMS score and sleep quality) from baseline to weeks 4, 12, and 24. We used Kruskal-Wallis equality-of-populations rank test to assess for between group differences in the outcome variables.

Univariable and multivariable linear regression analyses with robust standard errors were performed to primarily assess associations between log-transformed dolutegravir AUC_0-24_ estimates and outcomes (change in MMS score and sleep quality from baseline to weeks 4, 12, and 24), and secondarily assess genetic associations (*UGT1A1* rs887829 and *SLC22A2* rs316019) and the same outcomes. Separate regression models were performed at each of the three time points as evidence is lacking regarding the best time to assess for insomnia and NPAEs after starting dolutegravir. In the multivariable linear regression models, we adjusted for the following covariates selected *a priori*: baseline age, sex, CD4 T-cell count, log_10_ HIV-1 RNA, and nucleotide reverse transcriptase inhibitor (tenofovir disoproxil fumarate or tenofovir alafenamide).

## Results

Participant flow from the parent ADVANCE study for the primary and secondary objectives is shown in Supplemental Figures 2 and 3, respectively. Four-hundred-and-sixty-four participants were available for the primary analysis and 301 participants for the secondary analysis. Baseline characteristics of participants included in primary and secondary analyses are given in Table 1.

### Dolutegravir Pharmacokinetic-Pharmacodynamic Analyses

The median dolutegravir AUC_0-24_ estimate from participants in the tenofovir alafenamide (n = 231) and tenofovir disoproxil fumarate (n = 233) arms were 66.5 mg·h/L (interquartile range [IQR] 45.0 to 94.1) and 67.2 mg·h/L (IQR 54.0 to 95.3), respectively. The median dolutegravir AUC_0-24_ estimate from both arms was 66.7 mg·h/L (IQR 50.8 to 94.2).

### MMS Score

**Figure 1:**
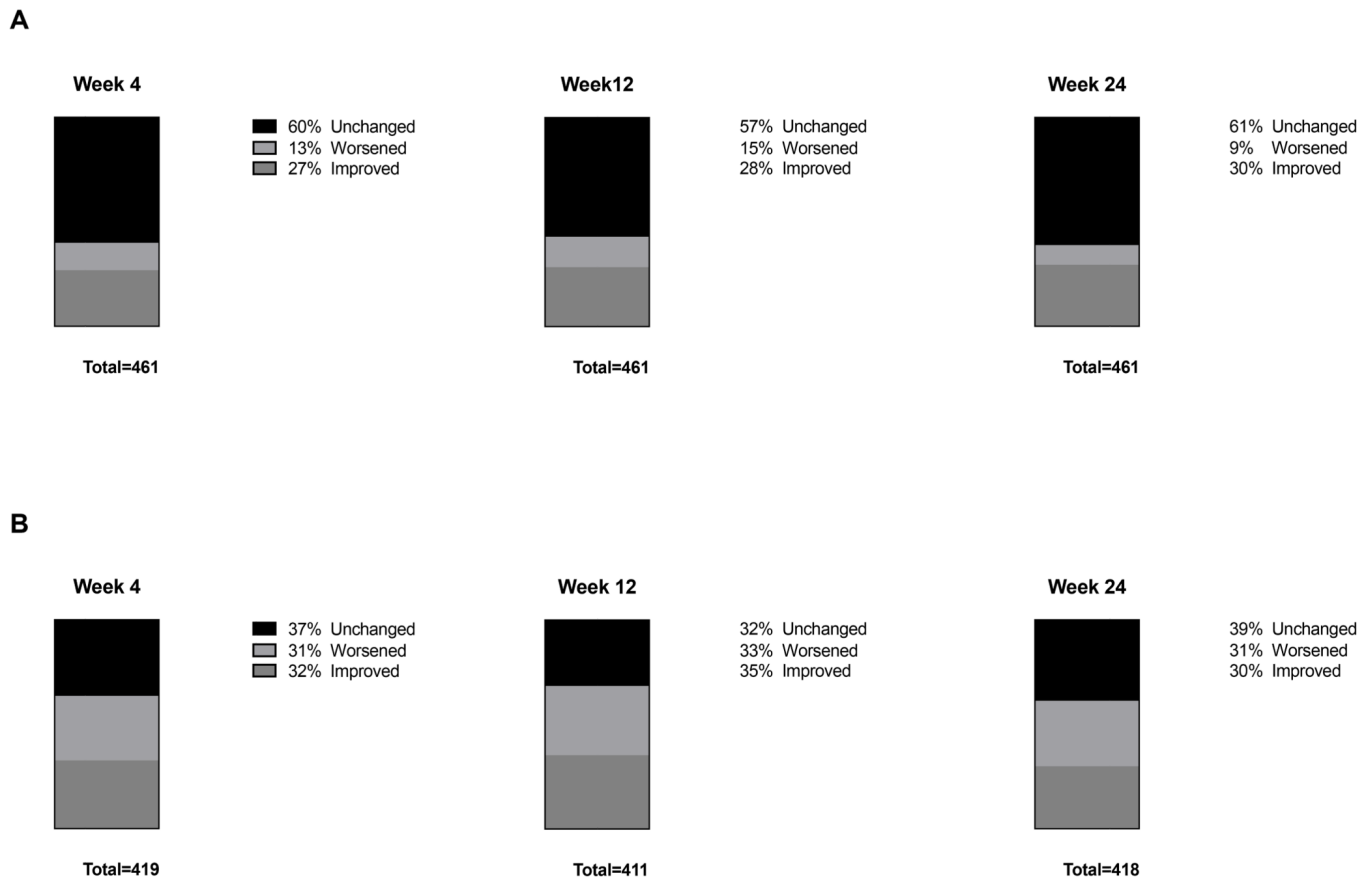
Stacked bar graphs representing percentage of participants in the primary analysis with unchanged, worsened, and improved modified mini screen score (A) and sleep quality (B) from baseline to week 4, 12, and 24.

The median change in MMS score from baseline to all 3 visits (week 4, 12, and 24) was 0 (IQR -1 to 0). The percentages of participants with unchanged, worsened, and improved MMS scores from baseline to weeks 4, 12, and 24 were similarly distributed between time points (Figure 1A).

Dolutegravir AUC_0-24_ was negatively correlated with change in MMS score from baseline to weeks 4, 12, and 24 (Table 2).

Univariable linear regression showed an inverse association between increasing dolutegravir AUC_0-24_ and change in MMS score from baseline to weeks 4, 12, and 24 (Supplemental Table 1). By multivariable linear regression there was a statistically significant inverse association between dolutegravir AUC_0-24_ and change in MMS score from baseline to week 12 and week 24 (Table 3), with higher dolutegravir exposure associated with more improvement from baseline in MMS.

### Sleep Quality

The median change in sleep quality from baseline to all 3 visits (week 4, 12, and 24) was 0 (IQR -1 to 0). The percentages of participants with unchanged, worsened, and improved change in sleep quality from baseline to weeks 4, 12, and 24 were similarly distributed between time points (Figure 1B). Dolutegravir AUC_0-24_ and change in sleep quality from baseline to weeks 4 and 12 were negatively correlated (Table 2).

Univariable linear regression showed an inverse association between increasing dolutegravir AUC_0-24_ and change in sleep quality from baseline to weeks 4, 12, and 24 (Supplemental Table 2). By multivariable linear regression the association was statistically significant only at week 12 (Table 4). Baseline CD4 count was independently associated with decreasing sleep quality from baseline to week 12 in univariable and multivariable analyses (Table 4).

### Genetic Analyses

The polymorphisms *UGT1A1* rs887829 and *SLC22A2* rs316019 were each in Hardy-Weinberg equilibrium. The minor allele frequency for *UGT1A1* rs887829 T allele was 41.6%, with genotype frequencies 34.2%, 48.4%, and 17.4% for CC, CT and TT, respectively. The minor allele frequency for *SLC22A2* rs316019 A allele was 12.5%, with genotype frequencies 76.3%, 22.4%, and 1.3% for CC, CA and AA, respectively.

Change in MMS scores from baseline to weeks 4, 12, and 24 were similar across genotypes. (Supplemental Figures 4, 5, and 6, respectively). There were no statistically significant associations between *UGT1A1* or *SLC22A2* genotypes and change in MMS score from baseline to week 4, 12, or 24 in either univariable or multivariable analyses (Supplemental Tables 1 and 3, respectively). Similarly, change in sleep quality scores from baseline to weeks 4, 12, and 24 were similar across genotypes (Supplemental Figure 4, 5, and 6, respectively). There were no significant associations between *UGT1A1* or *SLC22A2* genotypes and change in sleep quality from baseline to week 4, 12, or 24 in either univariable or multivariable analyses (Supplemental Tables 2 and 4, respectively).

## Discussion

We analysed data from a randomised controlled trial (the ADVANCE study) to characterise concentration-response relationships between dolutegravir and NPAEs. We found an independent association between worsening sleep quality from baseline to week 12 and increasing estimated dolutegravir AUC_0-24_. We found an unexpected independent association between more improved MMS scores from baseline to weeks 12 and 24 and increasing estimated dolutegravir AUC_0-24_, suggesting a positive effect of drug exposure on NPAEs. We did not find any associations between the selected *UGT1A1* rs887829 or *SLC22A2* rs316019 polymorphisms and change in MMS score or sleep quality from baseline to weeks 4, 12, or 24.

Studies have reported conflicting results on the association between dolutegravir exposure and NPAEs. Two cross-sectional studies, conducted in Japan and Italy, reported associations between dolutegravir exposure and NPAEs.^10,14^ Limited data from prospective longitudinal studies have not shown concentration-response relationships between dolutegravir and NPAEs. A small study (n = 40) failed to show associations between dolutegravir exposure and change in sleep assessment from baseline over 6 months.^11^ A population pharmacokinetic model derived from three phase 2 and 3 studies failed to show a relationship between dolutegravir exposure and the three most common adverse events (nausea, diarrhoea, and headache) but other NPAEs were not explored.^9^ Dolutegravir plasma concentrations were also not associated with risk for dolutegravir discontinuation secondary to observed NPAEs in a retrospective cohort of 37 participants.^12^

Our finding of improving MMS score with increasing dolutegravir exposure from baseline to weeks 12 and 24 was unexpected. Although these associations were statistically significant, the magnitude of effect was small as indicated by modest *r*_s_ values. Higher dolutegravir maximum concentrations were associated with improved global cognitive functioning in a prospective study,^11^ which is one possible explanation for our finding. Another possible explanation is that higher dolutegravir concentrations could be a marker for better adherence, and participants with NPAEs may have been less adherent.

Our finding that increasing dolutegravir exposure was associated with worsening sleep quality is consistent with other studies.^10,27^ Although only a change from baseline to week 12 in sleep quality was statistically significant for an association with increasing dolutegravir exposure, the *r*_s_ value for baseline to week 4 and multivariable linear regression effect coefficients for baseline to weeks 4 and 24 were in the same direction as the baseline to week 12 findings, regarding worsening sleep quality with increasing dolutegravir exposure.

We did not find associations between *UGT1A1* rs887829 or *SLC22A2* rs316019 and change in MMS score or sleep quality from baseline to weeks 4, 12, or 24. A Japanese study reported that *UGT1A1* loss-of-function polymorphisms, which result in higher dolutegravir exposure,^28^ were associated with a higher incidence of NPAEs in PLWH who switched to a dolutegravir-based regimen.^10^ An Italian study found an association between *SLC22A2* rs316019 and abnormal neuropsychiatric assessments among PLWH treated with a dolutegravir-containing ART regimen.^14^ However, the study was cross-sectional and could not assess baseline neuropsychiatric state prior to dolutegravir initiation. Our negative findings could be due to different genetic profiles among an African population or might be due to a lack of power to establish an association.

Our study has limitations. First, our findings in an ART-naïve African population may not be generalizable to other populations - only one participant in our sample was greater than 60 years of age, which is a reported risk factor for dolutegravir NPAEs.^8,29^ Second, we performed analyses of available data from the ADVANCE study, without any formal sample size calculation. However, our study is the largest to date to explore this relationship and NPAEs were elicited by repeated standardised questionnaires in the context of a randomised controlled trial. Third, our week 4 follow-up time point assessment may have missed earlier changes in NPAE. Efavirenz-related CNS side effects were more common in *CYP2B6* poor metabolisers at 2 weeks, but this difference was no longer present at 4 weeks.^30^ Fourth, the tools used for psychiatric and sleep quality assessment were subjective. Objective measures of sleep quality have proven superiority over subjective measures.^31,32^ Fifth, all participants in our study were ART naïve and this could potentially influence neuropsychiatric outcomes as mental wellbeing improves on treatment.^33-36^ An ART switch study might better assess concentration-response relationships between dolutegravir and treatment emergent NPAEs.^37^ Sixth, we did not assess headache or neurocognitive impairment in our study. Finally, we did not adjust for multiple statistical comparisons.

In conclusion, we found a concentration-response relationship between dolutegravir and worsening sleep quality (not statistically significant at weeks 4 and 24). We found an unexpected association of higher dolutegravir exposure with improving MMS score. We did not replicate previous genetic associations with dolutegravir-related NPAEs reported in two cross-sectional studies.^10,14^

## Supplementary Material

Supplementary material

## Figures and Tables

**Table 1:** Baseline characteristics of participants included in the primary analysis (assessment of associations between the outcome variables and dolutegravir AUC_0-24_ estimates) and secondary analysis (assessment of associations between the outcome variables and polymorphisms for *UGT1A1* rs887829 C→T and *SLC22A2* rs316019 C→A).

Baseline characteristics	Primary analysis (n=464)	Secondary analysis (n=301)
**Age**, years (median, IQR)	32 (27 – 38)	32 (27 – 38)
**Sex**		
Female, %	58.6	61.5
Male, %	41.4	38.5
**Race**		
Black, %	100	99.7
Mixed, %	0	0.3
**CD4 count**, cells/mm^3^ (median, IQR)	282 (165 – 442)	292 (161 – 457)
**HIV-1 RNA log_10_**, copies/mL (median, IQR)	4.4 (3.8 – 4.9)	4.4 (3.8 – 4.9)
**TAF/TDF arm**		
TAF, %	49.8	51.2
TFD, %	50.2	48.8
**MMS score**, (median, IQR)	0 (0 – 1)	0 (0 – 1)
**Sleep quality**, (median, IQR)	9 (8 – 10)	9 (8 – 10)

IQR=interquartile range, RNA= ribonucleic acid, TAF = tenofovir alafenamide, TDF = tenofovir disoproxil fumarate, MMS = modified mini screen

**Table 2:** Spearman’s rank-order correlations for dolutegravir AUC_0-24_ estimates and change in modified mini screen score and sleep quality form baseline to weeks 4, 12, and 24

	Spearman’s rank-order correlation for dolutegravir AUC_0-24_ and change in MMS form baseline				Spearman’s rank-order correlation for dolutegravir AUC_0-24_ and change in sleep quality form baseline			
Time Point	n	*r_s_*	95% CI	p-value	n	*r_s_*	95% CI	p-value
Week 4	461	-0.062	-0.153 to 0.029	0.186	419	-0.036	-0.127 to 0.056	0.468
Week 12	461	-0.099	-0.188 to -0.011	0.033	411	-0.101	-0.201 to -0.001	0.041
Week 24	461	-0.129	-0.222 to -0.036	0.006	418	0.002	-0.098 to 0.101	0.968

AUC_0-24_ = area under the concentration-time curve, MMS = modified mini score, *r_s_* = Spearman’s rank-order correlation

**Table 3:** Multivariable linear regression for change in modified mini screen score from baseline to week 4, week 12, and week 24 among participants with available estimated dolutegravir AUC_0-24_ concentrations

	Multivariable associations Week 4 (n=461)		Multivariable associations Week 12 (n=461)		Multivariable associations Week 24 (n=461)	
	Coefficient (95% CI)	p-value	Coefficient (95% CI)	p-value	Coefficient (95% CI)	p-value
Age (per 10 years increase)	-0.214 (-0.528 to 0.100)	0.181	-0.155 (-0.427 to 0.118)	0.265	-0.119 (-0.342 to 0.104)	0.296
Sex						
Female	Referent group					
Male	0.111 (-0.263 to 0.486)	0.559	0.167 (-0.173 to 0.507)	0.336	0.109 (-0.188 to 0.406)	0.472
Baseline CD4 count (per 50 cells/mm^3^increase)	-0.045 (-0.104 to 0.015)	0.141	-0.019 (-0.076 to 0.039)	0.521	-0.027 (-0.075 to 0.020)	0.258
Baseline HIV-1 RNA (per 1 log_10_ increase)	-0.140 (-0.394 to 0.114)	0.280	-0.133 (-0.394 to 0.127)	0.315	-0.115 (-0.324 to 0.094)	0.281
Arm						
TAF	Referent group					
TDF	-0.161 (-0.537 to 0.216)	0.402	-0.009 (-0.365 to 0.348)	0.962	-0.107 (-0.409 to 0.194)	0.486
DTG AUC_0-24_ (mg·h/L) (per 1 log_10_ increase)	-0.826 (-2.037 to 0.385)	0.181	**-1.278 (-2.250 to -0.306)**	**0.010**	**-1.145 (-1.953 to -0.338)**	**0.006**

TAF = tenofovir alafenamide, TDF = tenofovir disoproxil fumarate, DTG = dolutegravir, AUC_0-24_ = area under the concentration-time curve, PK = pharmacokinetics

**Table 4:** Multivariable linear regression for change in sleep quality from baseline to week 4, week 12, and week 24 among participants with available estimated dolutegravir AUC_0-24_ concentrations

	Multivariable associations Week 4 (n=419)		Multivariable associations Week 12 (n=411)		Multivariable associations Week 24 (n=418)	
	Coefficient (95% CI)	p-value	Coefficient (95% CI)	p-value	Coefficient (95% CI)	p-value
Age (per 10 years increase)	**-0.205 (-0.389 to -0.021)**	**0.029**	-0.142 (-0.319 to 0.035)	0.115	-0.043 (-0.228 to 0.142)	0.646
Sex						
Female	Referent group					
Male	0.060 (-0.235 to 0.354)	0.690	0.002 (-0.290 to 0.295)	0.987	-0.019 (-0.320 to 0.281)	0.899
Baseline CD4 count (per 50 cells/mm^3^ increase)	0.003 (-0.028 to 0.034)	0.871	**-0.030 (-0.059 to -0.001)**	**0.049**	-0.015 (-0.044 to 0.015)	0.338
Baseline HIV-1 RNA (per 1 log_10_ increase)	0.095 (-0.117 to 0.306)	0.379	-0.023 (-0.238 to 0.193)	0.837	0.069 (-0.124 to 0.261)	0.484
Arm						
TAF	Referent group					
TDF	0.205 (-0.078 to 0.487)	0.155	0.187 (-0.097 to 0.471)	0.197	0.023 (-0.266 to 0.313)	0.875
DTG AUC_0-24_ (mg·h/L) (per 1 log_10_ increase)	-0.552 (-1.343 to 0.239)	0.171	**-0.854 (-1.703 to -0.005)**	**0.049**	-0.096 (-0.854 to 0.663)	0.804

TAF = tenofovir alafenamide, TDF = tenofovir disoproxil fumarate, DTG = dolutegravir, AUC_0-24_ = area under the concentration-time curve, PK = pharmacokinetics

## Data Availability

The data that support the findings of this study are available from the corresponding author upon reasonable request.
