# Supplementary material for: Pharmacokinetic and Pharmacogenetic Associations with Dolutegravir Neuropsychiatric Adverse Events in an African Population
Source: J Antimicrob Chemother. Author manuscript; Available in PMC 2022 Oct 28. (PMC7613765; doi:10.1093/jac/dkac290)
Supplement: Supplementary material [file EMS154906-supplement-Supplementary_material_.docx]

**Supplemental Table 1:** Univariable linear regression for change in modified mini screen score from baseline to week 4, week 12, and week 24 among participants with available estimated dolutegravir AUC_0-24_ concentrations

|  | **Univariable associations**  Week 4 (n=461) | | **Univariable associations**  Week 12 (n=461) | | **Univariable associations**  Week 24 (n=461) | |
| --- | --- | --- | --- | --- | --- | --- |
|  | **Coefficient (95% CI)** | **p-value** | **Coefficient (95% CI)** | **p-value** | **Coefficient (95% CI)** | **p-value** |
| Age  (per 10 years increase) | -0.141  (-0.418 to 0.136) | 0.317 | -0.089  (-0.340 to 0.161) | 0.483 | -0.050  (-0.255 to 0.155) | 0.633 |
| Sex |  |  |  |  |  |  |
| Female | Referent group | |  | | Referent group | |
| Male | 0.113  (-0.253 to 0.480) | 0.545 | 0.157  (-0.195 to 0.510) | 0.380 | 0.112  (-0.186 to 0.410) | 0.460 |
| Baseline CD4 count  (per 50 cells/mm^3^ increase) | -0.034  (-0.088 to 0.021) | 0.227 | -0.014  (-0.067 to 0.038) | 0.591 | -0.023  (-0.065 to 0.019) | 0.281 |
| Baseline HIV-1 RNA  (per 1 log_10_ increase) | -0.001  (-0.230 to 0.229) | 0.996 | -0.051  (-0.287 to 0.184) | 0.668 | -0.014  (-0.186 to 0.159) | 0.878 |
| Arm  TAF  TDF | Referent group  -0.155  (-0.539 to 0.229) | 0.427 | Referent group  -0.0287  (-0.387 to 0.330) | 0.875 | Referent group  -0.123  (-0.426 to 0.180) | 0.426 |
| DTG AUC_0-24_ (mg·h/L)  (per 1 log_10_ increase) | -0.889  (-2.099 to 0.321) | 0.149 | **-1.255**  **(-2.250 to -0.261)** | **0.013** | **-1.199**  **(-2.030 to -0.368)** | **0.005** |
| *UGT1A1* rs887829 C→T | -0.014  (-0.412 to 0.385) | 0.947 | -0.220  (-0.574 to 0.133) | 0.221 | -0.049  (-0.355 to 0.257) | 0.754 |
| *SLC22A2* rs316019 C→A | -0.120  (-0.757 to 0.518) | 0.712 | 0.158  (-0.290 to 0.605) | 0.489 | 0.235  (-0.146 to 0.616) | 0.226 |

TAF = tenofovir alafenamide, TDF = tenofovir disoproxil fumarate, DTG = dolutegravir, AUC_0-24_ = area under the concentration-time curve, PK = pharmacokinetics

**Supplemental Table 2:** Univariable linear regression for change in sleep quality from baseline to week 4, week 12, and week 24 among participants with available estimated dolutegravir AUC_0-24_ concentrations

|  | **Univariable associations**  Week 4 (n=419) | | **Univariable associations**  Week 12 (n=411) | | **Univariable associations**  Week 24 (n=418) | |
| --- | --- | --- | --- | --- | --- | --- |
|  | **Coefficient (95% CI)** | **p-value** | **Coefficient (95% CI)** | **p-value** | **Coefficient (95% CI)** | **p-value** |
| Age  (per 10 years increase) | -0.171  (-0.354 to 0.011) | 0.066 | -0.090  (-0.266 to 0.086) | 0.314 | -0.021  (-0.202 to 0.160) | 0.821 |
| Sex |  |  |  |  |  |  |
| Female | Referent group | |  | |  | |
| Male | 0.080  (-0.210 to 0.371) | 0.587 | 0.056  (-0.236 to 0.350) | 0.705 | 0.020  (-0.236 to 0.350) | 0.896 |
| Baseline CD4 count  (per 50 cells/mm^3^ increase) | -0.005  (-0.030 to 0.021) | 0.727 | **-0.034**  **(-0.059 to -0.008)** | **0.009** | -0.019  (-0.045 to 0.007) | 0.151 |
| Baseline HIV-1 RNA  (per 1 log_10_ increase) | 0.090  (-0.092 to 0.273) | 0.330 | 0.067  (-0.124 to 0.259) | 0.490 | 0.105  (-0.071 to 0.282) | 0.240 |
| Arm  TAF  TDF | Referent group  0.185  (-0.102 to 0.473) | 0.206 | Referent group  0.185  (-0.104 to 0.474) | 0.209 | Referent group  0.024  (-0.263 to 0.311) | 0.869 |
| DTG AUC_0-24_ (mg·h/L)  (per 1 log_10_ increase) | -0.440  (-1.201 to 0.321) | 0.256 | **-0.864**  **(-1.685 to -0.044)** | **0.039** | -0.159  (-0.893 to 0.575) | 0.670 |
| *UGT1A1* rs887829 C→T | -0.109  (-0.361 to 0.142) | 0.393 | -0.154  (-0.401 to 0.092) | 0.219 | -0.036  (-0.267 to 0.195) | 0.760 |
| *SLC22A2* rs316019 C→A | 0.244  (-0.177 to 0.665) | 0.255 | 0.243  (-0.176 to 0.662) | 0.255 | 0.218  (-0.172 to 0.607) | 0.272 |

TAF = tenofovir alafenamide, TDF = tenofovir disoproxil fumarate, DTG = dolutegravir, AUC_0-24_ = area under the concentration-time curve, PK = pharmacokinetics

**Supplemental Table 3:** Multivariable linear regression for change in modified mini screen score from baseline to week 4, week 12, and week 24 among participants with available analysed genotypic polymorphisms

|  | **Multivariable associations**  Week 4 (n=299) | | **Multivariable associations**  Week 12 (n=301) | | **Multivariable associations**  Week 24 (n=298) | |
| --- | --- | --- | --- | --- | --- | --- |
|  | **Coefficient (95% CI)** | **p-value** | **Coefficient (95% CI)** | **p-value** | **Coefficient (95% CI)** | **p-value** |
| Age  (per 10 years increase) | -0.153  (-0.600 to 0.293) | 0.499 | -0.041  (-0.430 to 0.348) | 0.837 | 0.044  (-0.307 to 0.395) | 0.806 |
| Sex |  |  |  |  |  |  |
| Female | Referent group | |  | |  | |
| Male | 0.105  (-0.437 to 0.646) | 0.704 | 0.240  (-0.241 to 0.722) | 0.326 | 0.134  (-0.301 to 0.569) | 0.544 |
| Baseline CD4 count  (per 50 cells/mm^3^ increase) | -0.031  (-0.112 to 0.050) | 0.450 | 0.001  (-0.076 to 0.078) | 0.987 | -0.009  (-0.072 to 0.055) | 0.787 |
| Baseline HIV-1 RNA  (per 1 log_10_ increase) | -0.290  (-0.655 to 0.075) | 0.119 | -0.188  (-0.547 to 0.171) | 0.303 | -0.128  (-0.407 to 0.151) | 0.366 |
| Arm  TAF  TDF | Referent group  -0.269  (-0.853 to 0.314) | 0.364 | -0.073  (-0.594 to 0.449) | 0.783 | -0.295  (-0.741 to 0.151) | 0.194 |
| *UGT1A1* rs887829 C→T | -0.006  (-0.412 to 0.400) | 0.976 | -0.230  (-0.598 to 0.138) | 0.220 | -0.049  (-0.367 to 0.269) | 0.762 |
| *SLC22A2* rs316019 C→A | -0.079  (-0.712 to 0.554) | 0.806 | 0.187  (-0.260 to 0.634) | 0.410 | 0.247  (-0.132 to 0.625) | 0.201 |

TAF = tenofovir alafenamide, TDF = tenofovir disoproxil fumarate, DTG = dolutegravir, AUC_0-24_ = area under the concentration-time curve, PK = pharmacokinetics

**Supplemental Table 4:** Multivariable linear regression for change in sleep quality from baseline to week 4, week 12, and week 24 among participants with available analysed genotypic polymorphisms

|  | **Multivariable associations**  Week 4 (n=255) | | **Multivariable associations**  Week 12 (n=251) | | **Multivariable associations**  Week 24 (n=254) | |
| --- | --- | --- | --- | --- | --- | --- |
|  | **Coefficient (95% CI)** | **p-value** | **Coefficient (95% CI)** | **p-value** | **Coefficient (95% CI)** | **p-value** |
| Age  (per 10 years increase) | -0.241  (-0.498 to 0.017) | 0.067 | 0.015  (-0.235 to 0.265) | 0.904 | 0.060  (-0.172 to 0.292) | 0.611 |
| Sex |  |  |  |  |  |  |
| Female | Referent group | |  | |  | |
| Male | 0.209  (-0.160 to 0.579) | 0.266 | 0.115  (-0.269 to 0.498) | 0.556 | 0.200  (-0.166 to 0.567) | 0.283 |
| Baseline CD4 count  (per 50 cells/mm^3^ increase) | -0.006  (-0.047 to 0.034) | 0.750 | **-0.040**  **(-0.074 to -0.005)** | **0.023** | -0.012  (-0.050 to 0.025) | 0.522 |
| Baseline HIV-1 RNA  (per 1 log_10_ increase) | 0.098  (-0.158 to 0.354) | 0.450 | -0.028  (-0.262 to 0.207) | 0.818 | 0.163  (-0.068 to 0.394) | 0.167 |
| Arm  TAF  TDF | Referent group  0.248  (-0.128 to 0.625) | 0.194 | 0.158  (-0.225 to 0.541) | 0.417 | -0.024  (-0.385 to 0.338) | 0.898 |
| *UGT1A1* rs887829 C→T | -0.138  (-0.398 to 0.122) | 0.297 | -0.195  (-0.448 to 0.059) | 0.131 | -0.068  (-0.309 to 0.174) | 0.582 |
| *SLC22A2* rs316019 C→A | 0.278  (-0.145 to 0.702) | 0.197 | 0.287  (-0.150 to 0.725) | 0.197 | 0.229  (-0.167 to 0.624) | 0.256 |

TAF = tenofovir alafenamide, TDF = tenofovir disoproxil fumarate, DTG = dolutegravir, AUC_0-24_ = area under the concentration-time curve, PK = pharmacokinetics

**Supplemental Figure 1:** Modified mini score^1^


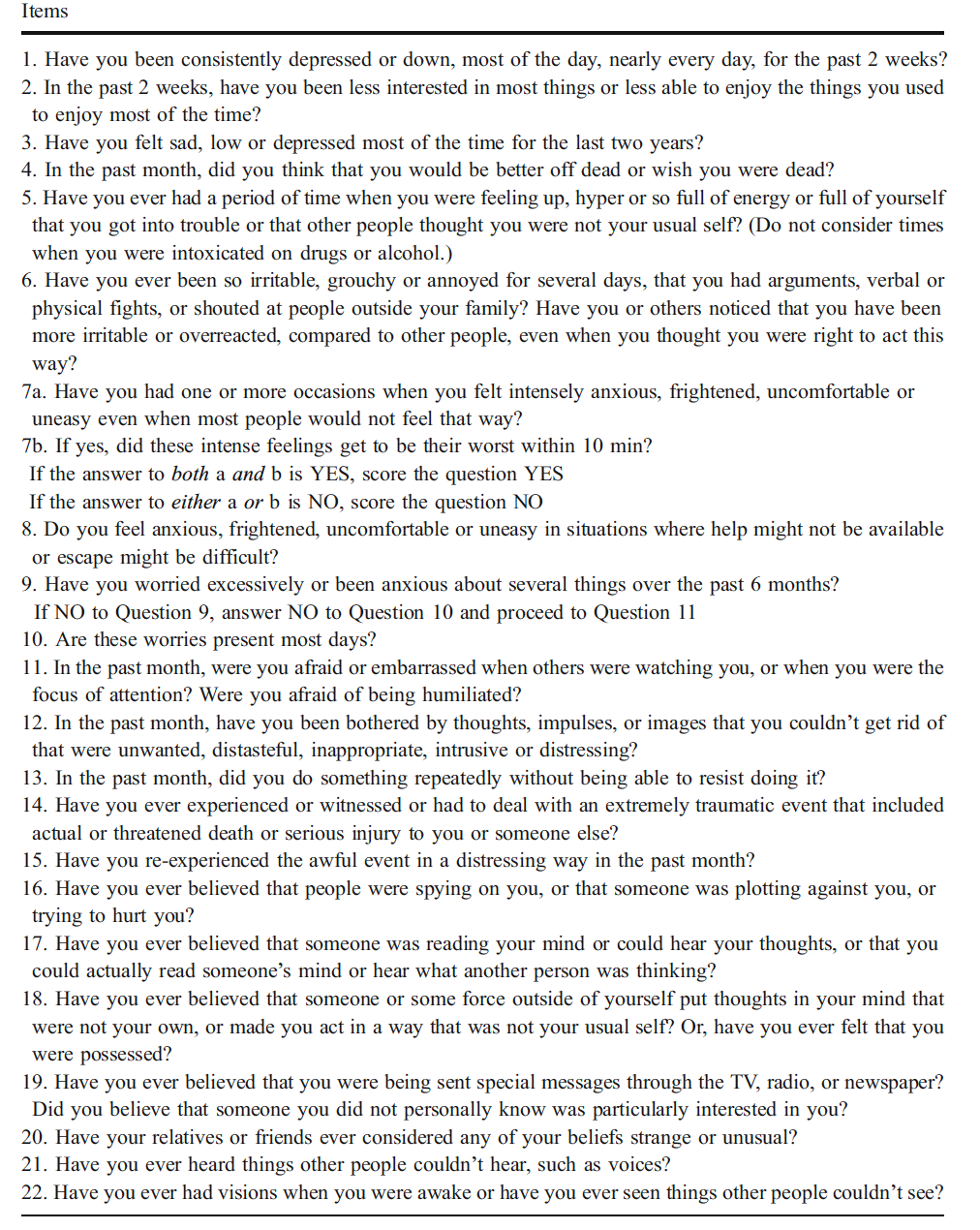


1. Alexander MJ, Haugland G, Lin SP, et al. Mental health screening in addiction, corrections and social service settings: Validating the MMS. Int J Ment Health Addict 2008; 6(1): 105–19.

**Supplemental Figure 2:** Participant flow diagram for primary analysis (FTC = emtricitabine, TAF = tenofovir alafenamide, TDF = tenofovir disoproxil fumarate, AUC_0-24_ = area under the concentration-time curve, MMS = modified mini screen)

**Supplemental Figure 3:** Participant flow diagram for secondary analysis (FTC = emtricitabine, TAF = tenofovir alafenamide, TDF = tenofovir disoproxil fumarate, AUC_0-24_ = area under the concentration-time curve, MMS = modified mini screen)

**Supplemental Figure 4:** Box plots of change in modified mini screen score from baseline to week 4 by (A) *UGT1A1* rs887829 C→T (Kruskal-Wallis equality-of-populations rank test P = 0.511) and (B) *SLC22A2* rs316019 C→A (Kruskal-Wallis equality-of-populations rank test P = 0.239) polymorphisms. Box plots of change in sleep quality from baseline to week 4 by (A) *UGT1A1* rs887829 C→T (Kruskal-Wallis equality-of-populations rank test P = 0.750) and (B) *SLC22A2* rs316019 C→A (Kruskal-Wallis equality-of-populations rank test P = 0.622) polymorphisms

A B

C D

**Supplemental Figure 5:** Box plots of change in modified mini screen score from baseline to week 12 (n = 301) by (A) *UGT1A1* rs887829 C→T (P = 0.11) and (B) *SLC22A2* rs316019 C→A (P = 0.84) status. Scatter plots of change in sleep quality from baseline to week 12 (n = 251) by (C) *UGT1A1* rs887829 C→T (P = 0.23) and (D) *SLC22A2* rs316019 C→A (P = 0.71) polymorphisms.

**Supplemental Figure 6:** Followed by box plots of change in modified mini screen score from baseline to week 24 by (C) *UGT1A1* rs887829 C→T (Kruskal-Wallis equality-of-populations rank test P = 0.092) and (D) *SLC22A2* rs316019 C→A (Kruskal-Wallis equality-of-populations rank test P = 0.754) polymorphisms. Followed by box plots of change in sleep quality from baseline to week 24 by (C) *UGT1A1* rs887829 C→T (Kruskal-Wallis equality-of-populations rank test P = 0.984) and (D) *SLC22A2* rs316019 C→A (Kruskal-Wallis equality-of-populations rank test P = 0.375) polymorphisms.

A B

C D
